# SUVmax of ^18^F-FDG PET/CT in the differential diagnosis of benign and malignant thyroid nodules according to tumor volume

**DOI:** 10.1186/s12957-015-0635-1

**Published:** 2015-07-16

**Authors:** Tae Heon Kim, Yong Bae Ji, Chang Myeon Song, Ji Young Kim, Yun Young Choi, Jeong Seon Park, Kyung Tae

**Affiliations:** Department of Otolaryngology–Head and Neck Surgery, College of Medicine, Hanyang University, 222 Wangsimni-ro, Seongdong-gu, Seoul 133-792 Korea; Department of Nuclear Medicine, College of Medicine, Hanyang University, Seoul, Korea; Department of Radiology, College of Medicine, Hanyang University, Seoul, Korea

**Keywords:** ^18^F-FDG PET/CT, Thyroid nodule, Thyroid cancer, Differential diagnosis, SUVmax

## Abstract

**Background:**

The aim of this study was to investigate whether the maximum standardized uptake value (SUVmax) measured on fluorine-18 fluorodeoxyglucose positron emission tomography/computed tomography (^18^F-FDG PET/CT) could be used as the primary means of differential diagnosis of thyroid nodules when tumor volume is assessed.

**Methods:**

We studied 192 patients who underwent preoperative ^18^F-FDG PET/CT followed by thyroidectomy. We evaluated the correlation between the volume of thyroid nodules, ^18^F-FDG uptake on visual analysis, and the mean SUVmax measured on ^18^F-FDG PET/CT.

**Results:**

When stratified by tumor volume, the mean SUVmax was higher in malignant than in benign nodules in nodules ≥1 cm^3^ (*p* < 0.001). However, it did not differ between benign and malignant nodules smaller than 1 cm^3^. At a cut-off value of SUVmax of 6, the respective sensitivities of ^18^F-FDG PET/CT, ultrasonography, and fine needle aspiration cytology were 60.8, 96.4, and 99.1 %, and the respective specificities were 95.9, 98.2, and 96.8 %.

**Conclusions:**

^18^F-FDG PET/CT is limited as a primary modality in the differential diagnosis of benign and malignant thyroid nodules because of its low sensitivity.

## Background

Unlike normal tissue, malignant tumors are characterized by increased glycolysis, which leads to elevated glucose uptake. Fluorine-18 fluorodeoxyglucose positron emission tomography/computed tomography (^18^F-FDG PET/CT) makes use of this to aid the diagnosis and staging of various human malignancies.

In recent years, ^18^F-FDG PET/CT has become widely used to diagnose thyroid cancer; it is especially useful in identifying sites of recurrence in patients with elevated serum thyroglobulin and negative whole body radioactive iodine scans after thyroidectomy for differentiated thyroid carcinoma [1–3]. ^18^F-FDG PET/CT also has potential advantages in the detection of distant metastasis or synchronous secondary tumor although its cost-effectiveness is not determined. However, the role of ^18^F-FDG PET/CT in the differential diagnosis of benign and malignant thyroid nodules is not yet well-established in clinical practice because some benign thyroid nodules show high ^18^F-FDG uptake, while some malignant nodules are not visualized at all. Consequently, there is a paucity of data regarding the role of ^18^F-FDG PET/CT in the differential diagnosis of thyroid nodules; the few studies that exist were conducted in small series of patients [4–6] or in patients whose nodules were identified incidentally during routine health checks [7–9]. In addition, while these studies estimated the mean maximum standardized uptake value (SUVmax) of ^18^F-FDG, they failed to consider the effect of nodule size. Other studies have enrolled patients with metastatic thyroid cancer or those with diffuse thyroid uptake of ^18^F-FDG.

Our aim in the present study was to establish whether the SUVmax measured on ^18^F-FDG PET/CT could be a primary modality in the differential diagnosis of benign and malignant thyroid nodules according to tumor volume.

## Methods

### Patients

We retrospectively studied 192 patients who underwent thyroidectomy for thyroid nodules and received a preoperative ^18^F-FDG PET/CT scan out of about 3500 patients who were diagnosed with thyroid nodule in our institute between January 2007 and December 2009. Of the tumors, 152 were malignant and 40 benign. Among the malignant cases, there were 146 papillary carcinomas, 3 follicular carcinomas, and 3 anaplastic carcinomas. The benign cases comprised 38 nodular hyperplasias and 2 follicular adenomas. Of the patients, 23 (15 %) were men and 129 (85 %) women. The mean age was 56 years, and all patients had normal thyroid function. The study protocol was approved by the Institutional Review Board of Hanyang University Hospital.

In this study, 141 patients with malignant nodule underwent ^18^F-FDG PET/CT scan to evaluate distant or regional metastasis after diagnosis of thyroid cancer by fine needle aspiration cytology (FNAC) according to the patient’s request or the recommendation of the doctors. Thirty-two patients with incidentally found benign and malignant nodule underwent ^18^F-FDG PET/CT scan for the purpose of routine health examination or evaluation of metastasis from other malignancy, and 19 patients of study group underwent ^18^F-FDG PET/CT for research purposes after submitting their written informed consent, especially in patients with follicular neoplasm or indeterminate cases on FNAC.

Exclusion criteria were recurrent thyroid cancer, diffuse ^18^F-FDG uptake by the thyroid that could not be differentiated from thyroid nodule on ^18^F-FDG PET/CT, and a history of thyroid surgery, or radiotherapy in the head and neck region for other diseases.

Patients with benign nodules underwent thyroidectomy: (1) for cosmetic purposes, (2) at their request, (3) if malignancy was suspected on ultrasonography (US), or (4) if their FNAC results were classified as inadequate or indeterminate.

Thyroid US and ultrasound-guided FNAC were performed by an experienced board-certified specialist in all cases. The FNAC results were categorized as (1) inadequate, (2) benign, (3) indeterminate (e.g., follicular neoplasm or atypia), (4) suspected malignancy, or (5) malignancy. In cases of microcalcification, irregular margins, marked hupoechogenity, or a taller-than-wide shape on US, the nodules were interpreted as malignant. Nodule diameter on US was used to calculate nodule volume. If there were multiple nodules on US, we measured a single nodule that was also examined by FNAC.

### ^18^F-FDG PET/CT

Whole body PET/CT images were acquired using a dedicated PET/CT system (Biograph 6, Siemens Medical Systems, Knoxville, TN, USA). Hard exercise was prohibited for several days, and the patients fasted for at least 6 h prior to the scan. Blood glucose levels were tested to confirm they were below 200 mg/dL. After an intravenous injection of ^18^F-FDG (0.21 mCi/kg), the patients were asked to wait for 1 h in a dimly lit room and encouraged to drink 1 L of water. A CT scan (80 mA and 140 kVp) was performed to correct attenuation prior to the PET scan. CT scanning was performed in 5-mm sections from the base of the skull to the proximal thigh; the images were reconstructed using a 512 × 512 matrix and a 50-cm field of view. PET scans were obtained from the proximal thigh to the base of the skull (six to seven bed positions, 2 min 30 s per each position), and the images were reconstructed with a 168 × 168 matrix, using the ordered subset expectation maximum iterative reconstruction algorithm, a 5-mm Gaussian filter, and a 50-cm field of view.

^18^F-FDG uptake by thyroid nodules was evaluated by visual analysis using the naked eye and quantitative measurement using SUVmax. If there is any focal increased uptake lesion detected in anterior neck on maximum intensity projection images by visual analysis, the uptake of thyroid nodule was verified by thorough examination of the axial PET/CT images by two nuclear medicine physicians. Then, SUVmax was measured for each thyroid nodule using volume of interest (VOI). SUV was calculated as follows: SUV = [FDG activity concentration (Bq/mL)] × [total lean body mass (kg)]/[injected FDG activity (Bq)].

### Statistical analysis

Statistical analyses were carried out with SPSS 17.0 (SPSS Inc., Chicago, IL, USA). We compared the uptake of ^18^F-FDG analyzed visually and SUVmax determined on ^18^F-FDG PET/CT between malignant and benign thyroid nodules. The same comparisons were made when the nodules were stratified by volume using a cut-off volume of 1 cm^3^. An independent Student *t*-test was performed to identify differences in SUVmax between the malignant and benign thyroid nodules. Pearson’s correlation coefficients were calculated to determine the correlation between nodule volume and the SUVmax.

## Results

### Differences in volume and SUVmax between malignant and benign nodules

The mean volumes of the malignant and benign nodules were not significantly different (11.63 ± 7.27 mL vs. 10.98 ± 4.56 mL; *p* = 0.968). On the other hand, the mean SUVmax was significantly higher in malignant than in benign nodules (5.11 ± 0.49 vs. 2.32 ± 0.25; *p* < 0.001). Among the malignant cases, the mean SUVmax was 4.80 ± 0.46 for papillary carcinoma, 17.66 ± 7.65 for follicular carcinoma, and 7.72 ± 3.44 for anaplastic carcinoma. In the benign cases, it was 2.45 ± 0.27 for nodular hyperplasia and 1.05 ± 1.05 for follicular adenoma.

### Differences in ^18^F-FDG uptake on visual analysis and SUVmax between malignant and benign nodules stratified by nodule volume

The differences between malignant and benign nodules in terms of visual ^18^F-FDG uptake and SUVmax, stratified by nodule volume, are shown in Tables 1 and 2. In nodules smaller than 1 cm^3^ (98 cases), ^18^F-FDG uptake on visual analysis was not different between the malignant and benign cases (*p* = 0.572). However, in nodules ≥1 cm^3^ (94 cases), it was significantly higher in malignant than in benign nodules (all patients with malignant nodules vs. 79.2 % of patients with benign nodules; *p* < 0.001) (Table 1). In nodules <1 cm^3^, mean SUVmax was 1.90 ± 0.21 in the malignant cases and 2.15 ± 0.42 in the benign cases (*p* = 0.630). However, it was significantly higher in malignant nodules with volumes ≥1 cm^3^ (8.88 ± 0.82 for malignant vs. 2.55 ± 0.33 for benign nodules, *p* < 0.001) (Table 2).

**Table 1:** ^18^F-FDG uptake by visual analysis of ^18^F-FDG PET/CT images in malignant and benign thyroid nodules according to nodule volume

Volume of nodule	Pathology	Visual analysis of ^18^F-FDG uptake	Number (%)	*p* value
Below 1 cm^3^ (*n* = 98)	Malignant nodule (*n* = 82)	Uptake	55 (67.1 %)	0.572
		No uptake	27 (32.9 %)	
	Benign nodule (*n* = 16)	Uptake	11 (68.8 %)	
		No uptake	5 (31.2 %)	
Equal to or above 1 cm^3^ (*n* = 94)	Malignant nodule (*n* = 70)	Uptake	70 (100 %)	<0.001
		No uptake	0 (0 %)	
	Benign nodule (*n* = 24)	Uptake	19 (79.2 %)	
		No uptake	5 (20.8 %)

**Table 2:** Mean SUVmax of malignant and benign nodules according to nodule volume

Volume of nodule	Pathology	Mean SUVmax	*p* value
Below 1 cm^3^ (*n* = 98)	Malignant nodule (*n* = 82)	1.90 ± 0.21	0.630
	Benign nodule (*n* = 16)	2.15 ± 0.42	
Equal to or above 1 cm^3^ (*n* = 94)	Malignant nodule (*n* = 70)	8.88 ± 0.82	<0.001
	Benign nodule (*n* = 24)	2.55 ± 0.33	

*SUVmax* maximum standardized uptake value of ^18^F-FDG PET/CT

### Correlation between nodule volume and SUVmax

There was no significant correlation between the volumes of all thyroid nodules and mean SUVmax (Pearson’s correlation coefficient, *r* = 0.149). Likewise, there was no significant correlation between the volume of malignant nodules only and mean SUVmax (*r* = 0.151). When stratified by volume, a weak correlation (*r* = 0.452) with SUVmax was observed in the case of small (<1 cm^3^) malignant nodules, but not in the case of larger (≥1 cm^3^) malignant nodules (*r* = 0.076).

### Diagnostic accuracy of ^18^F-FDG PET/CT, US, and FNAC

**Fig. 1:**
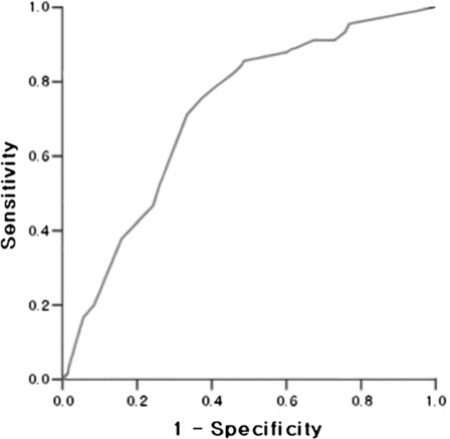
Receiver operating characteristic (ROC) curve of SUVmax. The diagnostic power of ^18^F-FDG PET/CT is the highest at SUVmax = 6

In the case of volumes ≥1 cm^3^, a receiver operating characteristic (ROC) curve analysis determined the optimal value of SUVmax for differential diagnosis between benign and malignant thyroid nodules (Fig. 1). The diagnostic power of ^18^F-FDG PET/CT was the highest at SUVmax = 6: at that value, it had 60.8 % sensitivity, 96.8 % specificity, a positive predictive value of 97.7 %, a negative predictive value of 48.4 %, and 70.2 % diagnostic accuracy.

We compared the results of thyroid US, FNAC, and ^18^F-FDG PET/CT at that cut-off value. Their respective sensitivities were 96.4, 99.1, and 60.8 %, and respective specificities were 95.9, 98.2, and 96.8 %. Similarly, their respective positive predictive values were 98.9, 99.4, and 97.7 %, and respective negative predictive values were 86.8, 95.2, and 48.4 %. Diagnostic accuracy was 97.3 % for thyroid US, 98.8 % for FNAC, and 70.2 % for ^18^F-FDG PET/CT.

### Diagnostic accuracy of ^18^F-FDG PET/CT in cases undiagnosed by FNAC

**Fig. 2:**
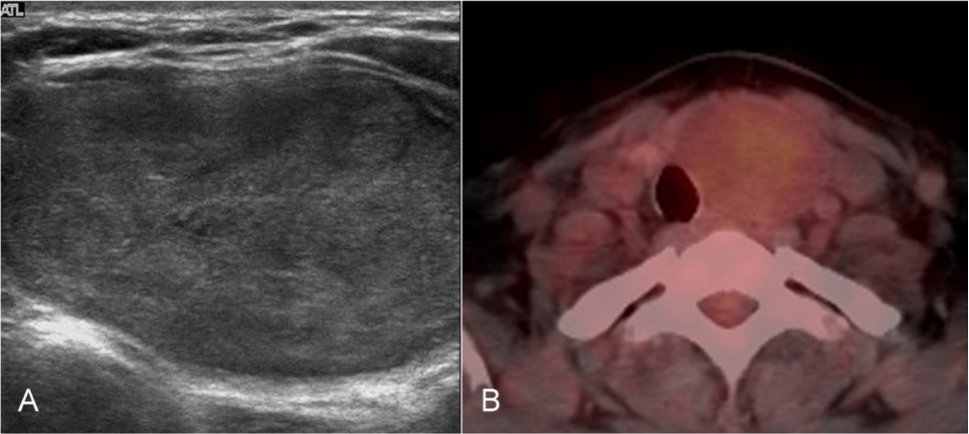
Images of follicular adenoma. **a** On ultrasonography, there is an 8-cm, markedly hypoechoic nodule. **b** Mildly increased ^18^F-FDG uptake in the left lobe on ^18^F-FDG PET/CT (SUVmax = 2.10)

**Fig. 3:**
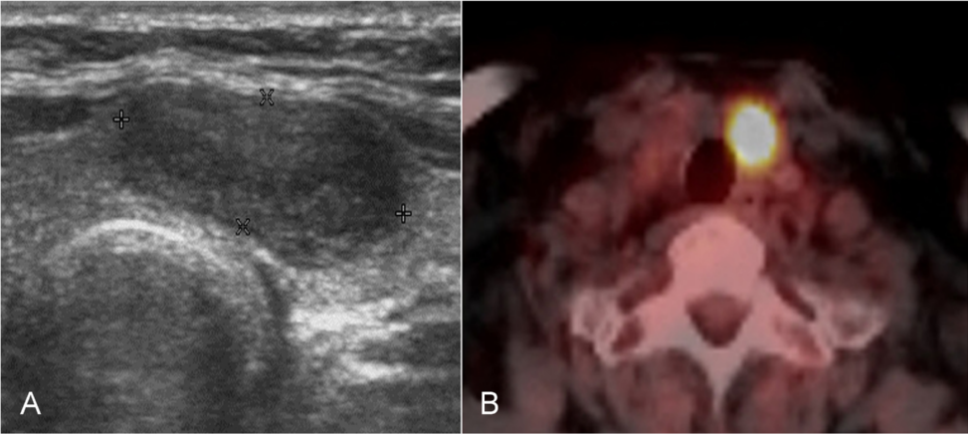
Images of follicular carcinoma. **a** On ultrasonography, there is a 1.7-cm, hypoechoic nodule. **b** Focal, intense ^18^F-FDG uptake in the left isthmic nodule on ^18^F-FDG PET/CT (SUVmax = 11.97)

FNAC diagnosed 151 cases of malignancy or suspected malignancy, 35 benign cases, 4 cases classified as indeterminate, and 2 as inadequate. Of the 151 supposed malignancies, 1 was found to be benign after thyroidectomy. Of the four indeterminate cases, two were confirmed as follicular carcinomas and two as follicular adenomas (Figs. 2 and 3). The cases classified as inadequate were found to be nodular hyperplasia (Table 3).

**Table 3:** Pathology results and SUVmax on ^18^F-FDG PET/CT in cases that were inconclusive on fine needle aspiration cytology

Results of FNAC	Group	SUVmax	Pathology
Indeterminate (n = 4)	Malignant (*n* = 2)	11.97	Follicular carcinoma
		8.2	Follicular carcinoma
	Benign (*n* = 2)	2.10	Follicular adenoma
		0	Follicular adenoma
Inadequate (n = 2)	Benign (*n* = 2)	2.6	Nodular hyperplasia
		0	Nodular hyperplasia

*FNAC* fine needle aspiration cytology, *SUVmax* maximum standardized uptake value of ^18^F-FDG PET/CT

When the six patients with indeterminate or inadequate findings on FNAC were examined by ^18^F-FDG PET/CT (with a cut-off SUVmax = 6), the method had 100 % sensitivity and specificity, 0 % false positives and false negatives, positive and negative predictive values of 100 %, and 100 % diagnostic accuracy.

## Discussion

In our study, the mean SUVmax was on the whole significantly higher in malignant than in benign thyroid nodules (*p* < 0.001). However, when the nodules were stratified by volume, we observed no significant differences in ^18^F-FDG uptake on visual analysis and in mean SUVmax between the malignant and benign nodules when the nodules were below 1 cm^3^. This might be due to the partial-volume effect, i.e., underestimation of small volumes. The resolution of ^18^F-FDG PET/CT is estimated at approximately 6 mm^3^ [10]. When ^18^F-FDG PET/CT is performed on smaller lesions, the adjacent normal tissue that does not show increased uptake is also present in the visual field, and this may lead to inaccurate assessment of FDG uptake. Hence, in this study, we evaluated the diagnostic accuracy of ^18^F-FDG PET/CT only in nodules with a volume ≥1 cm^3^ except for those nodules <1 cm^3^ in which neither visual analysis of uptake nor mean SUVmax had any power to differentiate between malignant and benign nodules.

As a tool in the differential diagnosis of benign and malignant thyroid nodules, thyroid US has been shown to have sensitivity of 30–75 %, specificity of 80–95 %, positive predictive value of 15–94 %, negative predictive value of 70–80 %, and diagnostic accuracy of approximately 75 % [11, 12]. By comparison, the sensitivity and specificity of FNAC have been estimated at approximately 85–90 % and 85–97 %, respectively, although these values may vary depending on the examiner’s technical skills [13, 14]. In our study, both thyroid US and FNAC had a higher diagnostic accuracy than suggested in the previous reports (the corresponding values for US in our study were 96.4, 95.9, 98.9, 86.8, and 97.3 %; and for FNAC: 99.1, 98.2, 99.4, 95.2, and 98.8 %). One of the contributing factors could be our study criteria, which excluded patients with negative findings on FNAC despite having intermediate findings or a suspected malignancy on US.

In the current study, the diagnostic ability of ^18^F-FDG PET/CT to differentiate benign and malignant thyroid nodules was lower than that of US or FNAC, even in larger (≥1 cm^3^) nodules. This implies that ^18^F-FDG PET/CT is less likely to be a primary modality in the differential diagnosis of all thyroid nodules. Furthermore, the role of ^18^F-FDG PET/CT as the primary modality in the differential diagnosis of thyroid carcinoma may be limited at the present time because the small thyroid cancer with a volume of <1 cm^3^ is common, and the diagnostic accuracy of ^18^F-FDG PET/CT is especially low in those cases. In addition, considering cost-effectiveness, ^18^F-FDG PET/CT is not recommended routinely.

However, the SUVmax of ^18^F-FDG PET/CT might be useful in the differential diagnosis of thyroid nodule detected incidentally by ^18^F-FDG PET/CT scan which was performed for the purpose of routine health examination or evaluation of metastasis from other malignancy. Our findings suggest that malignancy can be suspected when nodules ≥1 cm^3^ show FDG uptake on visual analysis of PET/CT images, because all the malignant thyroid nodules with a volume of ≥1 cm^3^ showed the uptake on visual analysis. In particular, there is a very high probability that thyroid nodules with a volume of ≥1 cm^3^ and SUVmax of >6 on ^18^F-FDG PET/CT are thyroid carcinoma because of a higher specificity of 96.8 % and a positive predictive value of 97.7 %.

According to reports, differential diagnosis by FNAC can be difficult in approximately 20 % of thyroid nodules, particularly in the case of non-diagnostic or unsatisfactory samples, follicular lesions of undetermined significance, or follicular neoplasms [15]. The diagnostic power of ^18^F-FDG PET/CT in such cases is still controversial [16–19]. Here, we evaluated the role of ^18^F-FDG PET/CT in six patients whose preoperative ultrasound-guided FNAC findings were not conclusively malignant or benign. In these patients, the sensitivity and specificity of ^18^F-FDG PET/CT were 100 %, indicating that it might be a useful modality in cases undiagnosed by FNAC. However, before these findings can be considered conclusive, they should be verified in a larger sample of patients.

Inevitably, this study has some limitations. The patients were not randomized, and there was selection bias. We included only those who underwent thyroidectomy and ^18^F-FDG PET/CT for benign or malignant nodules. We excluded any benign or indeterminate cases where thyroidectomy was not indicated and those cases in which ^18^F-FDG PET/CT was not performed.

## Conclusions

^18^F-FDG PET/CT is limited as a primary modality in the differential diagnosis of benign and malignant thyroid nodules because of its low sensitivity. However, malignancy can be suspected when a visual analysis of ^18^F-FDG PET/CT images shows increased FDG uptake in nodules with volumes ≥1 cm^3^. In addition, if SUVmax is greater than 6 in nodules ≥1 cm^3^, the probability of malignancy is very high.

## Abbreviations

^18^F-FDG PET/CT: fluorine-18 fluorodeoxyglucose positron emission tomography/computed tomography
FNAC: fine needle aspiration cytology
SUVmax: maximum standardized uptake value
US: ultrasonography
